# Side Effects and Efficacy of COVID-19 Vaccines among the Egyptian Population

**DOI:** 10.3390/vaccines10010109

**Published:** 2022-01-12

**Authors:** Marwa O. Elgendy, Ahmed O. El-Gendy, Sarah Mahmoud, Tarek Yehia Mohammed, Mohamed E. A. Abdelrahim, Ahmed M. Sayed

**Affiliations:** 1 Department of Clinical Pharmacy, Teaching Hospital of Faculty of Medicine, Faculty of Medicine, Beni-Suef University, Beni-Suef 62513, Egypt; Marwa.elgendy@nub.edu.eg; 2 Department of Clinical Pharmacy, Faculty of Pharmacy, Nahda University (NUB), Beni-Suef 62513, Egypt; 3 Department of Microbiology and Immunology, Faculty of Pharmacy, Beni-Suef University, Beni-Suef 62513, Egypt; ahmed.elgendy@pharm.bsu.edu.eg; 4 Department of Clinical and Chemical Pathology, Faculty of Medicine, Beni-Suef University, Beni-Suef 62513, Egypt; sara.mahmoud@med.bns.edu.eg; 5 Pharmacy Practice Department, Faculty of Pharmacy, Sinai University, El-Arish 45518, Egypt; tarek.yehia@su.edu.eg; 6 Clinical Pharmacy Department, Faculty of Pharmacy, Beni-Suef University, Beni-Suef 62513, Egypt; Mohamed.abdelrahim@pharm.bsu.edu.eg; 7 Department of Pharmacognosy, Faculty of Pharmacy, Nahda University, Beni-Suef 62513, Egypt; 8 Department of Pharmacognosy, Faculty of Pharmacy, AlMaaqal University, Basra 61014, Iraq

**Keywords:** BBIBP-CorV, ChAdOx1, BNT162, BioNTech, COVID-19 vaccine, vaccine antibodies, vaccine efficacy, vaccine side effects

## Abstract

Background: Knowledge about a vaccine’s side effects and efficacy is important to improving public vaccine acceptance. This study aimed to detect the safety and efficacy of vaccines among the Egyptian population. Methodology and Results: Data was collected using an online survey from participants who took two doses of the BBIBP-CorV, ChAdOx1, or BNT162 vaccines. Pain at the vaccine injection site, muscle pain, fatigue, dizziness, fever, and headache were the most common side effects after the first and second doses. The number pf side effects was higher in ChAdOx1 than in BNT162 and BBIBP-CorV. Most of the side effects started on the first day after vaccination and persisted for 1–2 days. Vaccinated people with past coronavirus infections before vaccination developed better antibodies than those who were only vaccinated. The side-effect severity was greater after the first dose of BBIBP-CorV and ChAdOx1 than after the second dose, but in contrast, the side-effect severity was greater after the second dose of BNT162 vaccine than after the first dose. ChAdOx1 was more effective than BBIBP-CorV, and one dose of ChAdOx1 produced an immune response similar to that of two doses of BBIBP-CorV. Conclusions: Coronavirus vaccines were well-tolerated, safe, and produced an immune response against the virus in most cases. Most postvaccine side effects were mild to moderate, which indicated the building of immunity by the body for protection.

## 1. Introduction

The onset of the COVID-19 pandemic and its rapid global spread harmed healthcare delivery worldwide [1,2,3]. However, the majority of infected people may be asymptomatic despite transmitting the infection. As a result, primary prevention at the community level is inherently difficult. The whole world has been trying to find a solution by vaccinating people [4] to eradicate the disease [5,6]. Coronavirus vaccines may protect people from getting infected with coronavirus or developing severe symptoms by motivating the immune system to produce antibodies [3,7,8,9]. After vaccination, the antibodies produced adhere to the invader spike protein and prevent the virus from gaining entry into the cells [10].

Four coronavirus vaccines are authorized for use all over the world: BNT162 (Pfizer BioNTech, New York, NY, USA), ChAdOx1 (AstraZeneca, Oxford, UK), mRNA1273 (Moderna, Cambridge, MA, USA), and Ad26.COV2-S (Johnson & Johnson, New Brunswick, NJ, USA). In addition, there are other vaccines, such as BBIBP-CorV (Sinopharm, Beijing, China), CoronaVac (Sinovac, Beijing, China), Sputnik V (Gamaleya, Moscow, Russian), and COVAXIN (Bharat Biotech, Hydrabad, India), which are authorized for use in many countries [9].

Building immunity after vaccination may sometimes cause side effects. These potential postvaccine side effects are considered the main cause of vaccine hesitancy among the population [11]. Increasing public awareness of the vaccine efficacy and being honest in clarifying the side effects are important to improving vaccine acceptance [11].

The side effects vary with the type of vaccine. Postvaccination side effects are more prevalent after vaccination with RNA (mRNA) than with other vaccines [12].

Most people develop immunity against coronavirus after vaccination, regardless of the absence or presence of side effects. A previous study showed that only one in four people suffered from mild and short-onset side effects after receiving coronavirus vaccines [13]. According to the World Health Organization, the most common side effects following coronavirus vaccines are fatigue, fever, headaches, pain at the injection site, nausea, and diarrhea [8]. This study was the first that aimed to determine the postvaccine side effects and efficacy of vaccines by measuring the level of antibodies against the coronavirus in the blood of vaccinated people by a quantitative IgG anti-spike-protein antibodies test.

## 2. Materials and Methods

### 2.1. The Study Design

The study was conducted from March to July 2021 in Egypt using an online questionnaire survey. The Research Ethical Committee of the Faculty of Medicine at Beni-Suef University authorized the study protocol (REC-FM BSU-15574) in accordance with the Declaration of Helsinki.

### 2.2. Sampling Technique

An online survey was designed to detect the postvaccine side effects. Healthy subjects who received two doses of BBIBP-CorV (Sinopharm), ChAdOx1 (AstraZeneca), or BNT162 (Pfizer BioNTech) vaccines participated in the study. A total of 168 participants participated in the study. The sample size was determined according to the previous report [14].

From the total participants, 78 participants recalled and agreed to undergo IgG anti-spike-protein antibodies tests after three days and three weeks after the first dose of vaccination with the BBIBP-CorV or ChAdOx1 vaccines, and three weeks after the second dose of vaccination with the BBIBP-CorV or ChAdOx1 vaccines.

### 2.3. Data Collection

Data were collected from the participants using a designated online questionnaire. The questionnaire was distributed over WhatsApp and Facebook using Google Forms. The questionnaire was circulated among Egyptians all over Egypt. The time needed to complete the questionnaire was 5 min. The questionnaire items were designed according to the information published by the FDA and the WHO.

The questionnaire was divided into three parts:

1. Participant demographic data: age, gender, educational level, marital status, current place of residence (country or city), and job;
2. Postvaccine first-dose side effects;
3. Postvaccine second-dose side effects.

### 2.4. IgG Anti-Spike-Protein Antibodies Test 

The results of the IgG anti-spike-protein antibodies test and laboratory tests were collected after three days and three weeks after first-dose vaccination with the BBIBP-CorV or ChAdOx1 vaccines, and three weeks after second-dose vaccination with the BBIBP-CorV or ChAdOx1 vaccines.

The test was carried out according to the previously reported immunoassay-based protocol [14] that was developed to measure IgG antibody titers in human serum or plasma. A chemiluminescent sandwich assay was used to run the test. The serum or plasma sample was initially exposed to SARS-CoV-2-specific recombinant antigens (Thermo Fisher Scientific, RP87680, Waltham, MA, USA) linked to magnetic beads in this technique. The antigen–antibody complex was treated with an alkaline phosphatase-conjugated antibody (Thermo Fisher Scientific, A-10648) against human IgG to generate a sandwich immunocomplex after bound/free separation. A luminous substrate was added to the solution after a second bound/free separation to enable luminescence measurement. Within 17 min of adding the substrate, the chemiluminescence intensity was measured at 450 nm. Throughout the procedure, the reaction chamber was kept at 42 °C. The concentrations of the samples were then calculated using the standard curve equation while taking into account the dilution of each sample [10].

## 3. Results

### 3.1. Characteristics of the Participants

A total of 168 (128 female) subjects participated in the study. Of them, 80 subjects received the BBIBP-CorV vaccine, 63 received ChAdOx1, and 25 received the BNT162 vaccine. Most of the participants were in the age range of 18 to 35 years old, married, city dwellers, and health care workers. The characteristics of the participants are shown in Table 1.

### 3.2. Postvaccine First-Dose Side Effects

#### 3.2.1. BBIBP-CorV Vaccine

Regarding the side effects after receiving the first dose of the BBIBP-CorV vaccine, the most common ones were pain, redness, or swelling at the site of vaccine injection (52.5%); fatigue and lethargy (45%); headache (15%); joint pain, muscle pain, and runny nose (10%); fever (7.5%); sore throat (6%); dizziness (5%); and cough, allergies, rashes, decreased appetite, and inflammation of the nervous system, including numbness, tingling, and loss of sensation (2.5%). However, 25% of the participants who received the BBIBP-CorV vaccine did not report any side effects. There were gap differences between BBIBP-CorV-vaccinated participants in answering the question, “To what extent do you rate the severity of these side effects?”; 49% answered that the side effects were mild, 18% answered that they were moderate, and 8% answered that they were severe. The majority of the BBIBP-CorV-vaccinated participants (67.5%) answered that the side effects appeared after the first dose, on the first day after receiving the vaccine. In addition, 25% of the BBIBP-CorV-vaccinated participants answered that the side effects that appeared after the first dose persisted for one day, 30% answered that they persisted for two days, and 20% answered that they persisted for more than two days. Regarding their postvaccine practices, 22.5% answered that they took pain relievers after taking the first dose of the vaccine, but 77.5% did not need to take any pain relievers.

#### 3.2.2. ChAdOx1 Vaccine

The most common side effects were pain, redness, or swelling at the site of vaccine injection (90.5%); muscle pain (71.5%); fatigue and lethargy (57%); joint pain (52%); fever and headache (38%); dizziness, abdominal pain, and convulsions and tremors (14%); inflammation of the nervous system, including numbness, tingling, and loss of sensation (13.5%); decreased appetite, nausea, and vomiting (9.5%); cough, allergies, rashes, and runny nose (5%); and sore throat (4.5%). Only 5% of the participants who received the ChAdOx1 vaccine did not feel any side effects, while 27% of the ChAdOx1-vaccinated participants answered that the side effects were mild, 54% answered that they were moderate, and 14% answered that they were severe. The majority of the ChAdOx1-vaccinated participants (76%) answered that the side effects appeared after the first dose during the first day after vaccination; 14% of the ChAdOx1-vaccinated participants answered that the side effects that appeared after the first dose persisted for one day, 28.5% answered that they persisted for two days, and 52.5% answered that they persisted for more than two days. Regarding their postvaccine practices, 81% answered that they took pain relievers after the first dose vaccination, but 19% did not need to take any pain relievers.

#### 3.2.3. BNT162 Vaccine

The most common side effects were pain, redness, or swelling at the site of vaccine injection (88%); fatigue and lethargy (50%); muscle pain and joint pain (20%); headache and runny nose (8%); and fever, dizziness, cough, allergies, rashes, convulsions, and tremors (4%). However, 8% of the participants who received the BNT162 vaccine did not feel any side effects; 32% of the BNT162-vaccinated participants answered that the side effects were mild, 50% answered that they were moderate, and 10% answered that they were severe. The majority of the BNT162-vaccinated participants (84%) answered that the side effects appeared after the first dose on the first day after vaccination; 64% of the BNT162-vaccinated participants answered that the side effects that appeared after the first dose persisted for one day, 16% answered that they persisted for two days, and 12% answered that they persisted for more than two days. Regarding their postvaccine practices, 28% answered that they took pain relievers after the first dose of vaccination, but 72% did not need to take any pain relievers. The postvaccine first-dose side effects are shown in Table 2.

### 3.3. Postvaccine Second-Dose Side Effects

#### 3.3.1. BBIBP-CorV Vaccine

Most of the participants who received the BBIBP-CorV vaccine received the second dose of the vaccine three weeks after the first dose, and answered that they were not infected with coronavirus between the first and second doses (or suffered from severe side effects of coronavirus for more than two days after receiving the first dose).

Regarding the side effects after receiving the second dose of the BBIBP-CorV vaccine, the most common ones were fatigue and lethargy (37.5%); pain, redness, or swelling at the site of vaccine injection (17.5%); headache, muscle pain, and runny nose (7.5%); sore throat, allergies, and rashes (5%); and joint pain, convulsions, and tremors (2.5%). On the other hand, 50% of the participants who received a second dose of the BBIBP-CorV vaccine did not report any side effects. There were differences in answering the question, “To what extent do you rate the severity of the side effects after the second dose?”: 32.5% answered that the postvaccine side effects were mild, and 17.5% answered that they were moderate. The majority of the participants (45%) answered that the postvaccine side effects appeared after the second dose on the first day after receiving the vaccine; 22.5% of the participants answered that the postvaccine side effects that appeared after the second dose persisted for one day, 15% answered that they persisted for two days, and 12.5% answered that they persisted for more than two days. Regarding their postvaccine practices, 17.5% answered that they took a pain reliever after taking the second dose of the vaccine, but 82.5% did not need to take any pain relievers.

#### 3.3.2. ChAdOx1 Vaccine

All of the participants who received the ChAdOx1 vaccine received the second dose of the vaccine three months after the first dose, and answered that they were not infected with coronavirus between the first and second doses.

Regarding the side effects after receiving the second dose of the ChAdOx1 vaccine, the most common ones were pain, redness, or swelling at the site of vaccine injection (82%); muscle pain (56%); fatigue and lethargy (48%); joint pain and fever (34%); headache (23%); decreased appetite (14%); and dizziness, nausea, and vomiting (5%). Only 14% of the participants who received the ChAdOx1 vaccine did not feel any side effects; 41% of the ChAdOx1-vaccinated participants answered that the side effects were mild, 40% answered that they were moderate, and 5% answered that they were severe. The majority of the ChAdOx1-vaccinated participants (80%) answered that the side effects appeared after the second dose on the first day after vaccination; 43% of the ChAdOx1-vaccinated participants answered that the side effects that appeared after the second dose persisted for one day, 27% answered that they persisted for two days, and 16% answered that they persisted for more than two days. Regarding their postvaccine practices, 55% answered that they took pain relievers after the second dose of vaccination, but 45% did not need to take any pain relievers.

#### 3.3.3. BNT162 Vaccine

All of the participants who received the BNT162 vaccine received the second dose of the vaccine three weeks after the second dose, and answered that they were not infected with coronavirus between the first and second doses.

Regarding the side effects after receiving the second dose of BNT162 vaccine, the most common ones were pain, redness, or swelling at the site of vaccine injection (92%); fatigue and lethargy (52%); fever (28%); joint pain (24%); muscle pain (20%); runny nose (8%); and dizziness, cough, allergies, rashes, convulsions, and tremors (4%). However, 8% of the participants who received the second dose of BNT162 vaccine did not feel any side effects; after the second dose of BNT162, 26% participants answered that the side effects were mild, 54% answered that they were moderate, and 12% answered that they were severe. The majority of the BNT162-vaccinated participants (84%) answered that the side effects appeared after the second dose on the first day after vaccination; 28% of the BNT162-vaccinated participants answered that the side effects that appeared after the second dose persisted for one day, 24% answered that they persisted for two days, and 40% answered that they persisted for more than two days. Regarding their postvaccine practices, 44% answered that they took pain relievers after the second dose of vaccination, but 66% did not need to take any pain relievers. The postvaccine second-dose side effects are shown in Table 3.

### 3.4. Results of IgG Anti-Spike-Protein Antibodies Test and Laboratory Tests at Day 18 after Vaccination with BBIBP-CorV or ChAdOx1 Vaccines

Regarding the participants who agreed to undergo an IgG anti-spike-protein antibodies test after three weeks of vaccination with BBIBP-CorV or ChAdOx1 vaccines, 16% of BBIBP-CorV-vaccinated participants had previously been infected with coronavirus during the four months before vaccination, and 23% of ChAdOx1-vaccinated participants had previously been infected with coronavirus during the four months before vaccination. A total of 51% of BBIBP-CorV-vaccinated participants showed negative results (less than 1 AU/mL) for the IgG anti-spike-protein antibodies test, and 49% showed positive results (more than 1 AU/mL) for this test. However, all of the ChAdOx1-vaccinated participants showed positive results for this test. The average positive results of quantitative IgG anti-spike-protein antibodies tests after three weeks of second-dose BBIBP-CorV vaccination for the cases who were infected with coronavirus during the four months before vaccination was 147 AU/mL, and for ChAdOx1 vaccination was more than 250 AU/mL. However, the average of positive results of quantitative IgG anti-spike-protein antibodies test after three weeks of second-dose BBIBP-CorV vaccination for the cases with no previous coronavirus infection before vaccination was 40 AU/mL, and for ChAdOx1 vaccination was 220 AU/mL.

The average positive results of quantitative IgG anti-spike-protein antibodies tests after three weeks of first-dose ChAdOx1 vaccination for cases infected with coronavirus in the four months prior to vaccination were 24 AU/mL.

Regarding the laboratory parameters for the positive IgG anti-spike-protein antibodies cases: average platelets were 263 × 109/L, average hemoglobin was 12.6 g/L, average leucocytes were 7.9 × 109/L, average neutrophils were 53%, average lymphocytes were 42%, average ESR was 21 mm/hr, average SGOT was 20 U/L, and average SGPT was 20.5 U/L.

The results of the IgG anti-spike-protein antibodies test and laboratory tests after vaccination with the BBIBP-CorV or ChAdOx1 vaccines are shown in Table 4.

## 4. Discussion

In this study, we demonstrated that the most common postvaccination side effects were pain, redness, or swelling at the site of vaccine injection; muscle and joint pain; fatigue and lethargy; dizziness; fever; and headache. We observed that the severity of side effects was greater after the first dose of BBIBP-CorV and ChAdOx1 vaccines than after the second dose, but in contrast, the severity of side effects was greater after the second dose of BNT162 vaccine than after the first dose.

Many vaccines have been developed during the past year. The vaccines must be safe and effective to be widely used in people to prevent the spread of the virus [15].

The knowledge about what happens after vaccination needs to be distributed among the general population. This may help to improve public education about coronavirus vaccines [7,16]. Fear and rumors, as well as a lack of clinical trial information, are considered factors that may lead to hesitancy in receiving the coronavirus vaccines [7]. The study questionnaire was structured based on the information published on the websites of the WHO and the FDA [17].

The items in the survey included postvaccine first-dose side effects and practices, postvaccine second-dose side effects and practices, and the results of IgG anti-spike-protein antibodies tests and laboratory after tests for first- and second-dose vaccinations.

The participants’ demographic data distribution showed a high percentage of respondents who were married, female, young, healthcare workers, and living in cities for those who received BBIBP-CorV or ChAdOx1 vaccines. This indicated that females were more concerned about their health and were afraid of infection, and they were encouraged to share their experiences with vaccines. The health care workers were more careful about being vaccinated due to the nature of their work and their usual exposure to COVID-19 patients to protect themselves from infection risk. This was also shown in the study by Rahul Shekhar et al. [18].

Most of the participants suffered from postvaccine side effects, which indicated that their immune systems did what they were supposed to do [19] Similar to what was shown in recently published studies, the most common postvaccination side effects were pain, redness, or swelling at the site of vaccine injection; muscle and joint pain; fatigue and lethargy; dizziness; fever; and headache. [9,16,20] Allergies and skin rashes can occur after vaccination. So, a person is prevented from getting vaccinated if they have a severe allergy (anaphylaxis) or an allergy to any ingredient in the vaccine. [21] Similar to the findings of a previous study [22], the BNT162vaccine was accompanied by anxiety during sleep.

It was noticed that 10% of BBIBP-CorV-vaccinated participants had been infected with coronavirus after receiving the first dose; however, for the ChAdOx1 and BNT162 vaccines, no infected cases were reported after receiving the first dose, which may indicate the higher immune response for ChAdOx1 and BNT162 vaccines (RNA- and DNA-based virus vectors) than for the BBIBP-CorV vaccine (inactivated virus). Overall, the decrease in the percentage of coronavirus-infected people after the first dose of vaccination may have been due to the higher percentage of health care workers who participated in this study; they are in a category with high education, and strictly follow preventive measures to avoid coronavirus infection [19].

As reported by the participants in this study, the post-BBIBP-CorV vaccine side effects after the first and second doses were commonly mild. Most of the participants reported that side effects after the first dose of ChAdOx1 were moderate, but after the second dose, the side effects were mild. In contrast to ChAdOx1, most of the participants reported that post-BNT162-vaccine side effects after the first dose were mild, but after the second dose, the side effects were moderate. The side effects were not critical and did not threaten life. These results were confirmed by previous studies [15,20]. Similar to the results of Qubais, B. et al. [20], it was observed that a higher percentage of BBIBP-CorV- and ChAdOx1-vaccinated participants did not have any side effects after the second dose of vaccination than after the first dose. That may have indicated that the severity of side effects was greater after the first dose of BBIBP-CorV or ChAdOx1 vaccines than after the second dose, but in contrast, the severity of side effects was greater after the second dose of BNT162 vaccine than after the first dose. A previous study also reported these results [22]. The Centers for Disease Control and Prevention showed that the intensity of side effects after the second dose of vaccination was greater than after the first dose [5].

All coronavirus vaccines are similar in their postvaccine side effects. Both the number of postvaccine side effects and their severity were associated with the nature and the mechanism of action of each vaccine. The number of side effects was higher in ChAdOx1 than in BNT162 than in BBIBP-CorV. This also was shown in a previous study by Ma’mon et al. [9].

Most of the participants had postvaccine side effects after the first dose, and these persisted for two days or more for the BBIBP-CorV vaccine, three days or more for the ChAdOx1 vaccine, and one day for the BNT162 vaccine. Regarding the post-second-dose vaccine side effects, they persisted for a day for the BBIBP-CorV and ChAdOx1 vaccines, and for three days or more for the BNT162 vaccine. That may have been related to the response of the immune system [20]. The immune system produces inflammatory mediators after vaccination, such as cytokines, which have inflammatory effects on body organs. Therefore, the post-coronavirus-vaccine side effects persist for days after taking the vaccine [3]. Most postvaccine side effects start during the first 24 h following vaccination and persist for 1–2 days [23].

The clinical trials conducted on Pfizer BioNTech (BNT162) showed that 50% of vaccinated people did not suffer from side effects, despite 90% of them developing immunity against the virus [23].

Most of the ChAdOx1-vaccinated participants used pain relievers, but most of the BBIBP-CorV- and BNT162-vaccinated participants did not use any pain relievers. This indicated that the severity of side effects after the ChAdOx1 first dose was greater than that which occurred after BBIBP-CorV and BNT162 first-dose vaccination. Most of the participants did not use any pain relievers after the second dose of BBIBP-CorV and ChAdOx1 vaccines, in contrast to most of the BNT162-vaccinated participants, who needed to use pain relievers. This indicated that the severity of side effects after BNT162 was greater than what occurred after BBIBP-CorV and ChAdOx1 second-dose vaccination. These side effects may have indicated that the body was building the desired immunity for protection [24].

It was obvious that post-BBIBP-CorV vaccine side effects after the first and second doses were commonly mild; therefore, a low percentage of participants needed to use pain relievers. Previous research has also found this [15,20]. After receiving the vaccine, the immune system produces sufficient amounts of antibodies to protect the body from coronavirus infection. Many people take nonsteroidal anti-inflammatory drugs (NSAIDs) to control the post-coronavirus-vaccine side effects. Some studies reported fears of taking NSAIDs to control the postvaccine side effects. NSAIDs cause inhibition of cyclooxygenase-1 (COX-1) and cyclooxygenase-2 (COX-2) enzymes, as well as inflammatory mediators such as cytokines. COX enzymes are important for sufficient antibody production after vaccination. Thus, using NSAIDs decreases the production of antibodies after coronavirus vaccination or infection [15,25].

The average laboratory parameters of the BBIBP-CorV- and ChAdOx1-vaccinated participants were normal, except for the erythrocyte sedimentation rate (ESR), levels of which were relatively elevated in vaccinated participants. An elevated level of ESR after coronavirus vaccination may be due to inflammation or false infection. This indicated that the vaccines did not affect the laboratory measures for vaccinated people, except for the inflammatory laboratory parameters. A study by Omar Tarawneh et al. showed similar results [26].

About 16% of the BBIBP-CorV-vaccinated participants and 23% of the ChAdOx1-vaccinated participants were previously infected with coronavirus in the last four months before vaccination. The antibody levels after coronavirus infection decreased over months, so the participants who were infected with coronavirus in the last four months before vaccination most likely still had postinfection antibodies that appeared in the postvaccine antibodies test.

Almost half of the BBIBP-CorV-vaccinated participants showed negative results for the IgG anti-spike-protein antibodies test after three weeks of second-dose vaccination, but all the ChAdOx1-vaccinated participants showed positive results. This difference in test results indicated that ChAdOx1 had a better ability to produce an immune response and sufficient antibodies than did BBIBP-CorV. Brazilian researchers also reported that Sinovac’s efficacy was 50.7% [27], while Turkish researchers reported an 83.5% efficacy for Sinopharm [28], and Indonesian researchers reported a 65% efficacy for Sinopharm [29].

Moreover, the average level of antibodies for ChAdOx1-vaccinated people was much higher compared with that of BBIBP-CorV-vaccinated people. This indicated that the ChAdOx1 vaccine had a higher efficiency, and can could a sufficient immune response that was better than that of the BBIBP-CorV vaccine.

After three weeks of the first dose of ChAdOx1 vaccination, the IgG anti-spike-protein antibodies test had positive results, but for the BBIBP-CorV vaccine, it had negative results. The average of positive results of the quantitative anti-spike-protein antibodies test (IgG) after three weeks of first-dose ChAdOx1 vaccination was close to the average of the positive results of the quantitative anti-spike-protein antibodies test (IgG) after three weeks of second-dose BBIBP-CorV vaccination. This indicated a higher efficacy of ChAdOx1 over BBIBP-CorV, and that one dose of ChAdOx1 produced an immune response similar to that of two doses of BBIBP-CorV. Another study reported a significant 39% drop in the rates of infection after 12–21 days of AstraZeneca (ChAdOx1) first-dose vaccination [22].

The vaccinated participants with past coronavirus infection reported satisfactory results for IgG anti-spike-protein antibodies, and tested better than those without past infection. The antibody concentrations were often higher in vaccinated people with past infections than in people who were only infected or only vaccinated. This may be explained by the fact that the body was dealing with the vaccine as if it were a second infection, so participants who were vaccinated after an infection had developed better immune responses to the vaccine. A previous study showed that only one dose of the vaccine was enough for people with past coronavirus infections to reach a satisfactory antibody level [27]. They suggested this topic to help solve the problem of the current vaccine shortage [27], as the global coronavirus vaccine production shortage is a large problem [27].

The elderly participants in this study experienced few postvaccine side effects, and reported low levels of antibodies in the antibodies test. This also was shown in a previous study [22,23]. A recently published study showed that postvaccine side effects were more common in younger people, and that the side effects were related to the process of building immunity, which is associated with postvaccine antibody production [16]. A previous study found that women suffered from side effects more than men, and explained that this may have been due to higher testosterone levels in men, which may contribute to the few side effects in men [22].

The strength of this study was that it was the first study to detect the side effects of the COVID-19 vaccine among Egyptians.

The limitations of this study were that, first, concerns about vaccine safety were a major obstacle to vaccination among Egyptians at the study time. Second, people younger than 18 years old were not included in the study, as they were not approved to take the vaccine at the time of the study.

## 5. Conclusions

Most of the side effects were mild to moderate, indicating that the body’s building of immunity was compromised. The severity of side effects was greater after the first dose of the BBIBP-CorV and ChAdOx1 vaccines than after the second dose, but in contrast, the severity of side effects was greater after the second dose of the BNT162 vaccine than after the first dose. ChAdOx1 was more effective than BBIBP-CorV, and one dose of ChAdOx1 produced an immune response similar to that of two doses of BBIBP-CorV. Vaccinated people with past coronavirus infections developed better immunity than those who were only vaccinated.

## Figures and Tables

**Table 1:** Characteristics of the participants.

Characteristic	BBIBP-CorV (%)	ChAdOx1 (%)	ChAdOx1 (%)
Gender			
Male	12.5%	19%	72%
Female	87.5%	81%	28%
Age (years)			
18 to 35 years	45%	47.5%	28%
35 to 45 years	32.5%	38%	40%
45 to 55 years	10%	4.5%	16%
55 years or more	12.5%	10%	16%
Social status			
Unmarried	24%	22%	16%
Married	76%	78%	84%
Current living location			
City	82%	79%	100%
Country	18%	21%	0%
Job			
I have no work	27.5%	6%	16%
I am a health care worker	55%	80%	32%
My job is in another field	17.5%	14%	52%

**Table 2:** Postvaccine first-dose side effects and practices.

Question	BBIBP-CorV (%)	ChAdOx1 (%)	BNT162 (%)
1—What are the post-vaccine first dose side affects you felt?			
Pain, redness, and swelling at site of vaccine injection	52.5%	90.5%	88%
Fever	7.5%	38%	4%
Sore throat	6%	4.5%	0%
			
Headache	15%	38%	8%
Muscle pain	10%	71.5%	20%
Excessive sweating	0%	0%	0%
			
Joint pain	10%	52%	20%
Dizziness	5%	14%	4%
Abdominal pain	0%	14%	0%
			
Decrease in appetite	2.5%	9.5%	0%
Nausea and vomiting	0%	9.5%	0%
Diarrhea	0%	0%	0%
			
Cough	2.5%	5%	4%
Allergies and skin rashes	2.5%	5%	4%
Runny nose	10%	5%	8%
			
Fatigue and lethargy	45%	57%	50%
Convulsions and tremors	0%	14%	4%
Swollen lymph nodes	0%	0%	0%
			
Blood coagulation	0%	0%	0%
Nervous system inflammation, including numbness, tingling, and loss of sensation	2.5%	13.5%	0%
I did not have any side effects	25%	5%	8%
2—To what extent do you rate the severity of these side effects?			
Mild	49%	27%	32%
Moderate	18%	54%	50%
Severe	8%	14%	10%
I did not have any side effects	25%	5%	8%
3—Side effects appeared after the first dose during:			
The first day after vaccination	67.5%	76%	84%
The second day after vaccination	2.5	19%	4%
The third day after vaccination	5%	0%	0%
After the third day from vaccination	0%	0%	4%
I did not have any side effects	25%	5%	8%
4—The side effects that appeared after the first dose continued for:			
A day	25%	14%	64%
Two days	30%	28.5%	16%
Three days or more	20%	52.5%	12%
I did not have any side effects	25%	5%	8%
5—You have received pain relievers			
Before receiving the first dose of the vaccine	0%	0%	0%
After receiving the first dose of the vaccine	22.5%	81%	28%
I did not use any pain relievers	77.5%	19%	72%

**Table 3:** Postvaccine second-dose side effects and practices.

Question	BBIBP-CorV (%)	ChAdOx1 (%)	BNT162 (%)
1—I received the vaccine second dose after			
Two weeks after the first dose	0%	0%	0%
Three weeks after the first dose	90%	0%	100%
Four weeks after the first dose	0%	0%	0%
More than four weeks after receiving the first dose	10%	100%	0%
2—Were you infected with coronavirus between the first and second doses (or you had severe symptoms of corona for more than two days after receiving the first dose)			
Yes	10%	0%	0%
No	90%	100%	100%
3—What are the post-vaccine second-dose side effects you felt?			
Pain, redness, and swelling at site of vaccine injection	17.5%	82%	92%
Fever	0%	34%	28%
Sore throat	5%	0%	0%
			
Headache	7.5%	23%	0%
Muscle pain	7.5%	56%	20%
Excessive sweating	0%	0%	0%
			
Joint pain	2.5%	34%	24%
Dizziness	0%	5%	4%
Abdominal pain	0%	0%	0%
			
Decrease in appetite	0%	14%	0%
Nausea and vomiting	0%	5%	0%
Diarrhea	0%	0%	0%
			
Cough	0%	0%	4%
Allergies and skin rashes	5%	0%	4%
Runny nose	7.5%	0%	8%
			
Fatigue and lethargy	37.5%	48%	52%
Convulsions and tremors	2.5%	0%	4%
Swollen lymph nodes	0%	0%	0%
			
Blood coagulation	0%	0%	0%
Nervous system inflammation, including numbness, tingling, and loss of sensation	0%	0%	0%
I did not have any side effects	50%	14%	8%
4—To what extent do you rate the severity of these side effects?			
Mild	32.5%	0%	26%
Moderate	17.5%	0%	54%
Severe	0%	5%	12%
I did not have any side effects	50%	14%	8%
5—Side effects appeared after the second dose during:			
The first day after vaccination	45%	80%	84%
The second day after vaccination	0%	6%	8%
The third day after vaccination	0%	0%	0%
After the third day from vaccination	5%	0%	0%
I did not have any side effects	50%	14%	8%
6—The side effects that appeared after the second dose continued for:			
A day	22.5%	43%	28%
Two days	15%	27%	24%
Three days or more	12.5%	16%	40%
I did not have any side effects	50%	14%	8%
7—You have received pain relievers			
Before receiving the second dose of the vaccine	0%	0%	0%
After receiving the second dose of the vaccine	17.5%	55%	44%
I did not take any pain relievers after receiving the second dose of the vaccine	82.5%	45%	66%

**Table 4:** Results of IgG anti-spike-protein antibodies test and laboratory tests after vaccination with BBIBP-CorV or ChAdOx1 vaccines.

Question		BBIBP-CorV	ChAdOx1
1—Have you had corona infection before?			
Yes			23%
No			77%
2—Results of anti-spike-protein antibodies test(IgG)			100%
Positive (more than 1)			100%
Negative (less than 1)			0%
3—Average of positive results of quantitative anti-spike-protein antibodies test (IgG) for the cases with a previous coronavirus infection during the four months before vaccination (after three weeks of second dose)			More than 250 AU/mL
4—Average of positive results of quantitative anti-spike- protein antibodies test (IgG) for the cases without previous coronavirus infection before vaccination (after three weeks of second dose)		40 AU/mL	220 AU/mL
5—Average of positive results of quantitative anti-spike-protein antibodies test (IgG) for the cases without previous coronavirus infection before vaccination (after three weeks of first dose)		0	24 AU/mL
6—Average of positive results of quantitative anti-spike-protein antibodies test (IgG) for the cases without previous coronavirus infection before vaccination (after three days of first dose)		0	0
7—Gender			
	Positive results of quantitative IgG anti-spike-protein antibodies test after second-dose vaccination		
	Male	54%	19%
	Female	46%	81%
	Negative results of quantitative IgG anti-spike-protein antibodies test after second-dose vaccination		
	Male	26%	0
	Female	74%	0
	Average of positive results of quantitative IgG anti-spike-protein antibodies test after second-dose vaccination.		
	Male	82 AU/mL	230 AU/mL
	Female	48 AU/mL	240 AU/mL
8—Age in years			
	Positive results of quantitative IgG anti-spike-protein antibodies test after second-dose vaccination		
	From 18 to 40 years	74%	51%
	From 41 to 60 years	20%	32%
	More than 60 years	6%	12%
	Negative results of quantitative IgG anti-spike-protein antibodies test after second-dose vaccination		
	From 18 to 40 years	44%	0
	From 41 to 60 years	34%	0
	More than 60 years	22%	0
	Average of positive results of quantitative IgG anti-spike-protein antibodies test after second-dose vaccination		
	From 18 to 40 years	72 AU/mL	250 AU/mL
	From 41 to 60 years	56 AU/mL	234 AU/mL
	More than 60 years	29 AU/mL	211AU/mL
9—Average of laboratory factors for the positive IgG anti-spike-protein antibodies cases			
Hemoglobin		12.6	12.5
Platelets		263	241.8
Leucocytes		7.9	6.7
Neutrophils		53%	59.6%
Lymphocytes		42%	32.4%
Erythrocyte sedimentation rate (ESR)		21	23
Aspartate aminotransferase (SGOT)		20	18.8
Alanine aminotransferase (SGPT)		20.5	18.9
